# Integrated Genomic Selection for Accelerating Breeding Programs of Climate-Smart Cereals

**DOI:** 10.3390/genes14071484

**Published:** 2023-07-21

**Authors:** Dwaipayan Sinha, Arun Kumar Maurya, Gholamreza Abdi, Muhammad Majeed, Rachna Agarwal, Rashmi Mukherjee, Sharmistha Ganguly, Robina Aziz, Manika Bhatia, Aqsa Majgaonkar, Sanchita Seal, Moumita Das, Swastika Banerjee, Shahana Chowdhury, Sherif Babatunde Adeyemi, Jen-Tsung Chen

**Affiliations:** 1 Department of Botany, Government General Degree College, Mohanpur 721436, India; dwaipayansinha@hotmail.com; 2 Department of Botany, Multanimal Modi College, Modinagar, Ghaziabad 201204, India; akmauryahrc@gmail.com; 3 Department of Biotechnology, Persian Gulf Research Institute, Persian Gulf University, Bushehr 75169, Iran; abdi@pgu.ac.ir; 4 Department of Botany, University of Gujrat, Punjab 50700, Pakistan; m.majeed@uog.edu.pk; 5 Applied Genomics Section, Bhabha Atomic Research Centre, Mumbai 400085, India; rachna@barc.gov.in; 6 Research Center for Natural and Applied Sciences, Department of Botany (UG & PG), Raja Narendralal Khan Women’s College, Gope Palace, Midnapur 721102, India; rashmimukherjee@rnlkwc.ac.in; 7 Department of Dravyaguna, Institute of Post Graduate Ayurvedic Education and Research, Kolkata 700009, India; gangulysharmistha23@gmail.com; 8 Department of Botany, Government, College Women University, Sialkot 51310, Pakistan; robina.aziz@gcwus.edu.pk; 9 TERI School of Advanced Studies, New Delhi 110070, India; manika.bhatia@terisas.ac.in; 10 Department of Botany, St. Xavier’s College (Autonomous), Mumbai 400001, India; aqsa.majgaonkar@xaviers.edu.in; 11 Department of Botany, Polba Mahavidyalaya, Polba 712148, India; sanchitaseal7@gmail.com; 12 V. Sivaram Research Foundation, Bangalore 560040, India; moumitataxonomy@gmail.com; 13 Department of Botany, Kairali College of +3 Science, Champua, Keonjhar 758041, India; swastikabiobot@gmail.com; 14 Department of Biotechnology, Faculty of Engineering Sciences, German University Bangladesh, TNT Road, Telipara, Chandona Chowrasta, Gazipur 1702, Bangladesh; shhnchowdhury00@gmail.com; 15 Ethnobotany/Phytomedicine Laboratory, Department of Plant Biology, Faculty of Life Sciences, University of Ilorin, Ilorin P.M.B 1515, Nigeria; adeyemi.sb@unilorin.edu.ng; 16 Department of Life Sciences, National University of Kaohsiung, Kaohsiung 811, Taiwan

**Keywords:** genomic selection, genomic gain, integrated genomic selection, marker-assisted selection, climate-smart cereals

## Abstract

Rapidly rising population and climate changes are two critical issues that require immediate action to achieve sustainable development goals. The rising population is posing increased demand for food, thereby pushing for an acceleration in agricultural production. Furthermore, increased anthropogenic activities have resulted in environmental pollution such as water pollution and soil degradation as well as alterations in the composition and concentration of environmental gases. These changes are affecting not only biodiversity loss but also affecting the physio-biochemical processes of crop plants, resulting in a stress-induced decline in crop yield. To overcome such problems and ensure the supply of food material, consistent efforts are being made to develop strategies and techniques to increase crop yield and to enhance tolerance toward climate-induced stress. Plant breeding evolved after domestication and initially remained dependent on phenotype-based selection for crop improvement. But it has grown through cytological and biochemical methods, and the newer contemporary methods are based on DNA-marker-based strategies that help in the selection of agronomically useful traits. These are now supported by high-end molecular biology tools like PCR, high-throughput genotyping and phenotyping, data from crop morpho-physiology, statistical tools, bioinformatics, and machine learning. After establishing its worth in animal breeding, genomic selection (GS), an improved variant of marker-assisted selection (MAS), has made its way into crop-breeding programs as a powerful selection tool. To develop novel breeding programs as well as innovative marker-based models for genetic evaluation, GS makes use of molecular genetic markers. GS can amend complex traits like yield as well as shorten the breeding period, making it advantageous over pedigree breeding and marker-assisted selection (MAS). It reduces the time and resources that are required for plant breeding while allowing for an increased genetic gain of complex attributes. It has been taken to new heights by integrating innovative and advanced technologies such as speed breeding, machine learning, and environmental/weather data to further harness the GS potential, an approach known as integrated genomic selection (IGS). This review highlights the IGS strategies, procedures, integrated approaches, and associated emerging issues, with a special emphasis on cereal crops. In this domain, efforts have been taken to highlight the potential of this cutting-edge innovation to develop climate-smart crops that can endure abiotic stresses with the motive of keeping production and quality at par with the global food demand.

## 1. Introduction

According to projections, the worldwide population is anticipated to increase by 2 billion individuals within the next three decades, resulting in a total of 9.7 billion people by the year 2050, up from 7.7 billion as recorded in 2019 [1]. The challenge of global food security is that by 2050, the world must feed two billion extra people, a quarter more than the current global population. Food demand will also increase by 56% from 2010 levels [2]. Food insecurity around the globe had increased to 828 million in the year 2021, an increase of 46 million from the previous year. Since the onset of the COVID-19 pandemic, 150 million more people have become food insecure [3]. There is a growing need for sustainable food production and nutrition, considering the rising pace of food insecurity. The 2020 census report indicates that 149 million children aged five years or younger were identified as stunted, while 45 million were classified as wasted. Additionally, 38.9 million children were observed to be overweight or obese globally [4]. 45% of fatalities in children (mostly under 5 years) were due to under-nutrition in low- and middle-income nations, where childhood obesity is also rising [4]. Food production must hence be increased, with a concomitant rise in the production of several essential commodities.

Apart from the rising population and increased food demand, climate change is also escalating food insecurity, threatening crop productivity and agricultural sustainability. The combination of an El Niño event and heat-trapping greenhouse gases will undoubtedly result in record-breaking global temperatures during the next five years. It is projected that there is a 66% risk of the annual mean near-surface global temperature surpassing pre-industrial levels by at least 1.5 °C for a minimum of one year during the period spanning from 2023 to 2027. This entire five-year span has a 98% risk of becoming the warmest [5]. Health, food security, water management, and the environment will all hence suffer [6].

Reduced energy demand owing to the societal and economic disruptions brought on by COVID-19 had decreased global carbon dioxide (CO_2_) emissions by 5.2% in 2020. However, as COVID-related limits were lifted throughout 2021, energy-related CO_2_ emissions increased by 6%, reaching a record high [7].

Furthermore, abiotic stress factors substantially influence the plant’s growth and yield. Plants encounter a range of climatic adversities in their native habitats, including waterlogging, drought, extreme temperatures, and exposure to salty air [8,9]. Ultraviolet-B (UV-B), light-intensity fluctuations, flooding, petrol emissions, heavy metals, and other physical and chemical components contribute to abiotic stresses [10]. In light of the current consumer trends, it is imperative to develop innovative strategies that utilize genetic enhancement to modify the production of staple crops to prevent significant damage to global food security within the next two to three decades [11]. Thus, to overcome these challenges, cultivars with increased yields are required.

Traditional breeding methods have yielded crops that are rich in nutrients and have high yields, which can be mechanically harvested to gratify the growing requirements for food among the population. Nonetheless, the at-present rate of yield enhancement for major crops, including wheat (*Triticum aestivum*), rice (*Oryza sativa*), and maize (*Zea mays*), is inadequate to satisfy future demand [12]. Also, to provide for the growing human population, crop plants’ production potential must increase while their yield gaps must be shrink [13,14]. Modern crop-breeding and management techniques have contributed significantly to an annual increase of 0.8–1.2% in agricultural yield. However, the present pace of genetic progress is inadequate to satisfy the demands of the projected global populace by the 2050s [11,15]. It is challenging to attain the required rate of genetic advancement using standard breeding techniques, particularly in light of soil degradation and shrinking usable water resources under the influence of climate change. Additionally, mostly all yields and agronomic qualities are very much genetically complicated and heavily impacted by external influences, making it challenging to improve them using traditional breeding techniques [16].

Thus, to resolve the issues of global food demand in conjunction with climate change, the induction of climate-smart agriculture is the necessity of the hour. Climate-smart agriculture (CSA) is a comprehensive approach to landscape management that encompasses cropland, livestock, forests, and fisheries and seeks to rationalize the effects of climatic alteration while ensuring food security. CSA has set its sights on concurrently accomplishing three goals: increasing productivity, increasing the adaptability to abiotic and biotic stressors, and reducing emission patterns, thereby protecting the environment [17]. Climate-smart agriculture has the potential to improve food security in several ways, such as increasing crop output, decreasing the likelihood of so-called crop failure, and mitigating the negative effects of climate change [18]. Climate-smart crops form the backbone of climate-smart agriculture and can offer resilience to extreme environmental conditions. In addition, they can efficiently meet the growing food demand through increased productivity. The recent developments in sequencing technologies have ushered in a new era in crop breeding and caused a revolution in genetics [19].

**Figure 1:**
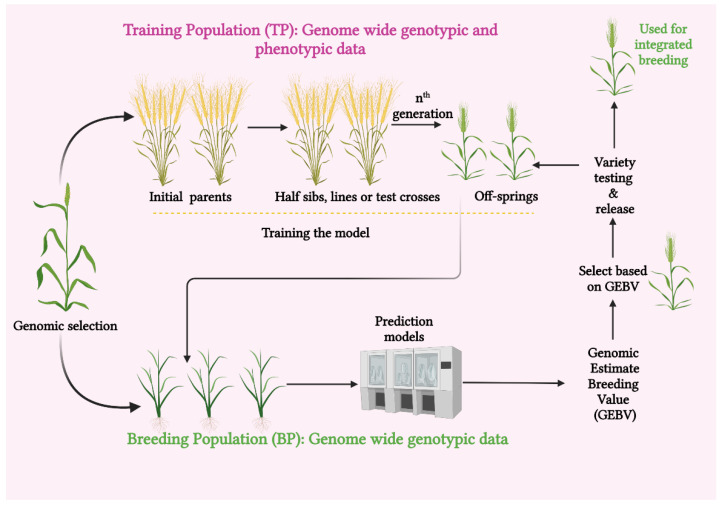
Schematic representation of steps involved in genomic selection (created with BioRender.com).

One of the versatile crop-breeding selection methods based on gene-sequencing technology is genomic selection. Genomic selection analyses the variation within a crop population using gene-sequencing techniques [20]. The GS was first put forward by Lande and Thompson in 2000 [21] and popularized by Meuwissen et al. (2001) [22]. Genomic selection is described as a subset of marker-assisted breeding (MAB), in which every quantitative trait locus (QTL) is checked to be in linkage disequilibrium (LD) with at least one genetic marker. This ensures an efficient selection of the desired traits. This strategy is now more practical due to the large number of single nucleotide polymorphisms (SNPs) that have been ascertained by genome sequencing and the development of new techniques that efficiently genotype large numbers of SNPs [23]. Genomic selection has many advantages, including reducing the need for extensive field testing and hastening the transmission of genetic gain with shorter generation intervals and at a reduced cost. Assessing both additive and non-additive genetic variances are also among its potential advantages [24] (Figure 1).

Moreover, the breeding cycle is accelerated through GS, making it easier to identify superior genotypes rapidly [25]. Genomic selection is an identified promising approach for enhancing complex trait genetics with a substantial reduction in the breeding cycles. However, the routine compounding of GS into crop-breeding programs necessitates the refinement of GS models to account for genotype–environment interaction (GEI) and non-additive effects, as well as for the reduction of associated costs. This can be accomplished by integrating GS with high-throughput genotyping and phenotyping platforms and with speed breeding. These platforms make it easier to increase the speed along with the accuracy of the GS-assisted breeding process and also to produce better genetic gains per unit of time and expense [16]. Genomic selection enables quick crop improvement without the need for an in-depth analysis of individual loci. A phenotyped and genotyped training population (TP) is used to anticipate the genomic estimated breeding values (GEBVs) of specific lines in GS. Without the additional time-consuming phenotyping, a breeding population (BP) can be established from the selected individuals and bred over several generations [26]. Focusing on cereal crops, this review aims to emphasize GS methods and practices and the realms connected with them. To maintain output and quality with the global food demand, efforts have been made to highlight the outlook of this so-called cutting-edge technology to evolve climate-smart crops that can withstand abiotic challenges.

## 2. Methodology

This review intends to systematically and comprehensively integrate all the relevant, scientific, and policy information that are available in relation to climate change, population census, food demand, integrated genomic selection, climate-smart crops (CSCs), and incidental material with special reference to abiotic stresses. The review has been split into parts such as CSCs, integrated genomic selection strategy, the importance of genetic gain, and the benefit of GS that forms the basis of preferring IGS over other breeding approaches, The review also highlights the opportunities and challenges associated with IGS vis-à-vis climate-smart crops.

The review incorporates the information in an integrative mode and the available information was pooled by retrieving papers from web-based resource depositories such as PubMed, Google Scholar, Web of Science, Scopus, and individual journal platforms. The information was collected by using keywords like genomic selection, integrated genomic selection, omics approaches for genomic selections, genomic selection + climate smart cereals, integrated genomic selection + cereal, climate-smart crops, climate-smart crops + cereals, speed breeding, approaches of plant breeding, climate change + impacts on cereals, population growth + food demand, sequencing technologies, etc. Recent papers were selected on priority except for the pioneering work, which required due mention in the manuscript.

## 3. Climate-Smart Crops: A Promising Option for Future Food Security

**Figure 2:**
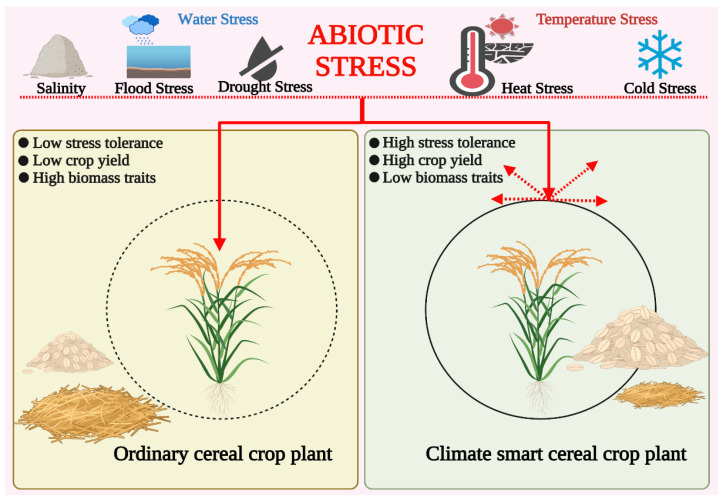
Comparative depiction of traits exhibited by ordinary and climate-smart cereal crop plants in response to abiotic stress (created with BioRender.com).

The rising population and rapidly changing climate pose many challenges and risks to our ecosystem. Climate change has affected ecosystem productivity and is expected to reduce staple crop yield by 30% [27,28,29]. Plant breeding has been critical in fulfilling rising demand since crop domestication. Nonetheless, it has not proven sufficient so far and has been surpassed by modern molecular breeding procedures and advanced integrated genomics methods [30]. But, as seen in the recent past, climate change effects are worsening. It is, therefore, pertinent to develop climate-resilient crops or climate-smart crop (CSC) varieties to practice climate-smart agriculture (CSA) [31]. These CSCs are equipped with the required traits to tolerate multidimensional stresses with optimum crop yield and efficient crop biomass regulation [32]. Crop biomass may both produce and remove greenhouse gases (GHGs); hence, managing crops through CSA can help with sustainable development [33] (Figure 2). Global institutions like the Food and Agriculture Organization (FAO), World Bank, International Monetary Fund (IMF), and International Center for Agricultural Research in the Dry Areas (ICARDA) are openly advocating the development and adoption of CSCs providing tolerance against abiotic and biotic stress [17,34,35,36].

## 4. Integrated Genomic Selection for Making Climate-Smart Cereals

**Figure 3:**
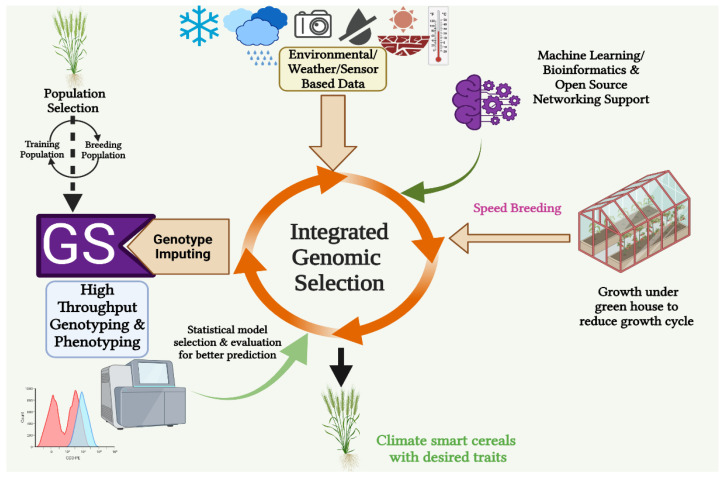
Diagrammatic representation of different components of integrated genomic selection methods used for developing climate-smart cereal crops (created with BioRender.com).

The issue of climate change has gained widespread recognition as a significant challenge. The decline in yields of key food crops has resulted in an expansion of the demand–supply gap for the worldwide population [37]. Thus, developing stress-resilient crops has become inevitable and a research priority [38]. After the successful implementation of GS in the animal system, plant breeding has also recognized and adopted it as a potential breeding tool for rapid and superior genotype selection with minimum breeding time, as gene-sequencing-technology-dependent marker-assisted selection was not efficient in capturing all the favorable and economically valuable alleles during crop improvement [39]. Further improvements were made by integrating GS with high-throughput phenotyping (HTP), genotyping, and machine-learning (crop/eco-physiological modeling) and speed-breeding methods. Such integration is cumulatively called integrated genomic selection (IGS) [30]. It enables breeders to execute GS in larger breeding programs with multifarious utility in situations where genome sequences are available. Integrated genomic selection results show greater accuracy of GS and a high genetic gain per unit of time with reduced expenditure [16]. In Asian rice (*O. sativa* L.), GS demonstrated improved prediction accuracy when used in conjunction with a phenological model to forecast the emergence date of untested genotypes in untested conditions [40]. Sensor-based phenomics used in the IGS approach beyond biomass for varietal protection (VP) and nutritive traits (NT) facilitated the expansion of perennial ryegrass (*Lolium perenne* L.) breeding [41] (Figure 3).

Similarly, greenhouse phenotyping integrated with GS can help to deliver early selection with moderate accuracy. It can be applied in conjunction with reproductive techniques that shorten the generation time and help to accelerate genetic gains, as seen in a population of *Populus deltoides* [42]. Incorporating environmental data sourced from past weather databases or forecasted through climate change models into the analysis of crop models can yield valuable metadata regarding phenology. This information can aid in investigating the target population of environments (TPEs), providing enhanced insights into current and future TPEs. Additionally, this approach can facilitate the improved design of phenotype testing [43]. The potential of GS and integrating “omics” data for disease evaluation in wheat has also been reviewed [44,45]. Researchers have also emphasized that GS approaches from dairy cattle breeding cannot be easily applied to complex plant-breeding programs [46]. Genomic selection relies on additive genetic effects to predict genetic outcomes, and it can therefore enhance genetic progress and crop enhancement by incorporating a wide range of previously unexplored genetic diversity sources as non-additive effects [11] to make IGS more meaningful. The genotype–environment interaction or QTL inheritance makes it difficult to increase grain yield in breeding and improvement programs, including in wheat breeding [47,48]. However, IGS was found to be a valuable decision-making tool in identifying genotypes [30,49]. It could be combined with breeders’ observation, germplasm knowledge, and experience associated within and beyond a breeding program to harness optimum output, as observed for simultaneous selection to develop superior wheat varieties for grain output and protein content as well as the dough rheological traits related to baking quality [50]. To implement an integrated multi-trait breeding strategy with a sizable hybrid maize population, the target-oriented priority (TOP) machine-learning approach has been proposed. The study determined that the method is dependable and sturdy and is anticipated to offer assistance in making breeding choices during an extensive search for germplasm that exhibits high yield and resilience to climate variability [51].

## 5. Genetic Gain: A Metric for Tracking Breeding Initiatives’ Forward Development

The term “genetic gain”, or “genetic gain from selection” refers to the increase in the average genetic value or average phenotypic value of a population brought about by selection within that group during generations of breeding. Following is the way to estimate genetic gain [52]:

ΔG = i σAIrMG/t,(1)

ΔG = the predictable genetic gain

i = the power of selection

σA = genetic SD, or the square root of additive genetic variance

rMG = selection(2)

t = breeding cycle time(3)

It is possible to enhance the genetic gain for a given period by reducing the breeding cycle gap “t”. The creation of cultivars of crops with greater nutritional density and climatically adaptable features, both of which are necessary for a sustainable food supply, would be accelerated by rates of genetic gain in crop-breeding programs [53]. Grain output and food security can be increased in the context of environmental changes through the use of genetic gain [53]. Genomic selection, which chooses candidates for the upcoming breeding cycle based on individuals’ GEBVs gathered from genome-wide markers, is a viable method for improving quantitative traits. Genomic estimated breeding value is a method useful in plant breeding to assess a plant’s genetic potential for specific features. It is based on DNA markers. A breeding program using GS has two steps.

(1) Genotyping and phenotyping of entities in a reference population and the building of a statistical format to study the effects of SNPs on morphological makeup, creating relational forecasting equations.
(2) Newer candidates might not be phenotyped but are genotyped. Additionally, breeding values are calculated using phenotypic data and prediction models [54]. Owing to its increased genetic gain, reduced phenotyping, shorter cycle times, and improved selection accuracy, GS has been warmly accepted in breeding programs around the world over the past two decades. The feasibility of using GS in breeding crops is also being looked into, as it has given promising early evaluation results in the betterment of yield, biotic and abiotic stress resilience, and, of course, quality in cereal crops [55].

## 6. The Benefits of Genomic Selection over Conventional Approaches and Marker-Assisted Selection

Phenotypic behaviors of crop plants are the pivotal characteristics on which the entire plant breeding and management approach is based. Classical plant breeding techniques rely on morphological or phenotype-based (marker) selection methods [56,57]. The accurate prediction of phenotype is difficult because it is governed by many loci and by the result of genotype–environment interactions [58,59]. Phenotypic selection methods are indirect and inefficient as they are easily influenced by environmental factors and the growth cycle (gene actions) [58,60]. In the quest to improve crop productivity, various other types of selection methods were evolved over phenotypic selection in modern plant breeding systems, such as cytological, biochemical, and molecular markers/DNA markers [61,62]. Cytological markers reflect the variations present in terms of chromosome number, size, shape, order, position, and banding patterns as well as differences in the distribution of euchromatin and heterochromatin. These variations help in the differentiation between wild and mutated chromosomes, linkage group identification, and physical mapping [63]. Biochemical markers are represented by isozymes encoded by different genes having similar functions, and they reveal allelic variations of an enzyme that help to estimate gene and genotypic frequencies [64,65]. These properties are applied for the identification of genetic diversity, gene flow, population structure, and subdivision. Biochemical markers are co-dominant, user-friendly, and economically feasible, but because of their lower number, low polymorphism and tedious extraction methodologies limit their utility [66]. To overcome the problems encountered in phenotypic, cytological, or biochemical marker-based selection methods, collectively termed as pre-DNA-marker methods [67], modern molecular (DNA) MAS methods were developed. Beckmann and Soller, in 1986, first used the term “marker-assisted selection” [68,69]. This was an outcome of the advancement of molecular biology techniques that gave further insights into the nucleotide sequence polymorphisms generated due to insertions, deletions (InDel), duplications, point mutations, and translocations [66]. Amplified Fragment-Length Polymorphisms (AFLP), Inter-Simple Sequence Repeat (ISSR), Random Amplified Polymorphic DNA (RAPD), Restriction-Fragment-Length Polymorphism (RFLP), SNP, Simple Sequence Repeat (SSR), Diversity Arrays Technology (DArT), and retrotransposons are some of the examples of molecular markers routinely used in agriculture. The application of molecular markers differs from species to species [70]. These molecular markers are mostly co-dominant, but some are dominant. They are widely replicable and uniformly dispersed across the genome. As the associated marker has a strong connection to the gene of interest, the MAS may simply deduce the existence or omission of a gene by examining the marker [71]. Also, the molecular markers remain uninfluenced by environmental factors and agricultural plant growth conditions, making them a better choice over pre-DNA markers [61]. In a breeding program, molecular markers like MAS offer an important edge over traditional markers for selecting and screening crop plants [72]. The four primary uses of molecular markers in crop breeding [73] are:

*(a)*  *To overcome the limitations of conventional phenotypic selection*

Traits that are difficult to be improved by conventional phenotypic selection such as low penetrance or complex inheritance can be improved by molecular markers as the former selection method is time-consuming.

*(b)*  *Freedom of choice of selection at a specific stage*

Selection at specific environmental and developmental stages is helped by MAS, as applied in excluding two biotic problems of Cassava genetic improvement, namely the assortment for resistance to CMD in Colombia and whitefly disease.

*(c)*  *Helpful for backcross breeding*

MAS is used for speeding up backcrossing for the maintenance of recessive alleles where molecular markers help the linkage drag, a challenging phenomenon to remove all unwanted genes.

*(d)*  *Pyramiding multiple monogenic traits*

Pyramiding indicates the process where multiple genes/QTLs are combined into a single genotype. It is helpful for those traits that involve several QTLs for a single target trait with multipart inheritance, namely pest and disease resistance or quality traits. Although traditional or classical breeding can also be used to create pyramids, this is exceedingly difficult or impossible during the early generations. The molecular markers clear all these obstacles of conventional breeding and facilitate the pyramiding process.

Apart from the above advantages, MAS can be performed by obtaining material directly from plant sources, ranging from seed tissue to any stage or part of the plant, making it useful to work with at any stage. It also gives additional opportunities to work at certain crucial stages when specific traits are expressed, such as pollen development to study male sterility, study of grain sensitivity to photoperiod, or fruit quality [74]. Moreover, pre-flowering genetic data made available through MAS makes it possible to regulate pollination [61]. Although MAS presents multiple advantages, it has its own set of limitations as well. The high cost, sophisticated laboratory ecosystem, need for trained resource persons, use of hazardous radioisotopes, and time-consuming procedure to carry out RFLP, RAPD, AFLP, SNP analyses, etc. are some of its disadvantages. QTL studies are the crucial limitation of MAS due to their cumulative effects as they are influenced by environmental and genetic interactions [75]. However, after the invention of PCR, the study of molecular markers has become fast, cost-effective, and efficient [73,76].

**Figure 4:**
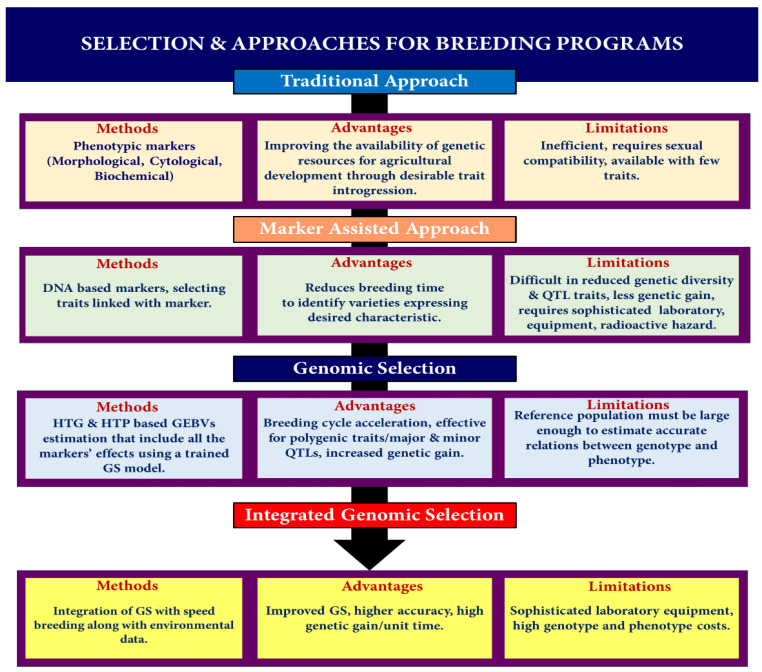
Flow chart showing features and limitations of various approaches ranging from classical to modern integrated genomic selection applied in plant breeding.

A variation of the MAS technique that utilizes SNP markers is known as GS. These markers span an organism’s whole genome. The number of impacts per QTL that must be estimated is minimal because the markers are expected to be in LD related to the QTL. According to simulation studies, the breeding value may be anticipated with a precision of 0.79 to 0.85 [23,77]. A study indicated the impact of marker density and type (microsatellite and SNP) on accuracy and found that a SNP requires 2 to 3 times the density of microsatellites to obtain comparable accuracy [77]. This is because SNPs are biallelic while microsatellites are multiallelic in nature. Similarly, an accuracy comparison of the predicted breeding value using four different models by estimating the total breeding value by GS vis-à-vis pedigree information obtained from conventional selection confirmed that GS is more accurate, especially for low-heritability traits, indicating that GS has an advantage even at low marker densities [78]. Earlier, the chief limitation of GS was the high cost of genotyping a large number of markers. The development of modern cost-effective, rapid, and efficient sequencing technologies like next-generation sequencing (NGS) and developments in SNP genotyping technology [79,80] have obviated the cost factor, and now, many plant genomes have been sequenced and are available [81]. Sequencing data obtained from advanced sequencing technologies are combined with high-throughput phenotyping (HTP), which enables genome-wide association studies (GWAS) and SNP identification within complex genetic architectures [82,83]. Researchers can use high-throughput phenotyping to collect data on a wide variety of phenotypes from a large number of individuals or samples, which can then be paired with sequencing data to explore the association between genetic variations (SNPs) and phenotypic traits. The combination of HTP and sequencing data increases the strength and scope of genome-wide association analyses, making it easier to identify the genetic factors underlying complex traits [84]. It has been suggested that GS has great potential when integrated with somatic embryogenesis (SE) incorporated with multivarietal forestry (MVF), as it captures both additive and non-additive variations and eliminates the time required for seed production, thereby causing higher genetic gains per unit time [85]. Hence, it is earnestly required to adopt similar models for creating climate-smart cereals. GS has several merits over other MAS approaches, including the elimination of the requirement for significant field testing, increased transmission of genetic gain, and shortened generation intervals that make the quick identification of superior genotypes feasible [25]. The inclusion of both additive and non-additive genetic variance assessments in GS gives additional potential benefits [24]. These advantages have been used in the situation of asexually propagated crops, where non-additive effects hinder preference and influence the efficacy of the substances under assessment since they cannot be passed effectively onto the next cycle of selection. Recurrent selection schemes can be accelerated since the GS assesses numerous loci, haplotypes, or marker impacts over the whole genome to calculate the GEBV, allowing farmers to exploit all the genetic influences in the production field [22,86], which can be used to accelerate the genetic gain in asexually propagated crops such as cassava [87] (Figure 4) (Table 1).

## 7. Genomic Selection Methodology

### 7.1. Design of Training Population

Genomic selection possesses the ability to considerably boost the genetic improvement rates in plant breeding programs. It offers the advantage of estimating all marker effects simultaneously, resulting in greater gains from selection [55]. Unlike traditional MAS, which relies on a limited number of significant markers, GS identifies genetically superior individuals based on their GEBV. This broader approach allows for more comprehensive selection and has the potential to bring about significant advancements in plant breeding programs [91].

The problem of association mapping and QTL discovery is finding and measuring uncommon genetic markers with delicate impacts on economically relevant traits that are heavily impacted by environmental influences. To address this difficulty, statistical models for predicting the link between markers and traits are being created, taking into consideration the genetic makeup of the particular feature of concern [92]. The introduction of high-density SNP arrays with several thousand markers aided in the creation of these models [25]. However, when it comes to genomic prediction (GP) and forecasting traits in non-phenotyped individuals within specific settings (such as site-year combinations), the inclusion of gene–environment interactions in the statistical models introduces significant complexity in the GP models, resulting in lower accuracy [25]. To address this complexity, statistical–genetic models should incorporate multiple traits and multiple environments and consider the genetic correlations and variance–covariance between environments, traits, and their interactions. To untangle the intricacies of multi-trait genomics and multiple settings, a theoretical framework that takes into account these deep interactions is required [93]. Overall, studies have consistently demonstrated high prediction accuracy, which highlights the value of GS as a plant breeding strategy. However, compared to animals, predicting plant traits across breeding cycles presents greater challenges [94]. The phenotypic response is defined by GP models as the combination of genetic values (depicted by linear additive versions) and a residual value. Extensive research in GP has been devoted to constructing parametric and nonparametric statistical and computational models that enhance the precision of forecasting non-phenotyped genotypes [93].

Advances in GS and GP have increased data volume and complexity. This has inspired multidisciplinary research endeavors that combine various fields such as computer science, quantitative genetics, genetics, mathematics, physics, statistics, machine learning, and bioinformatics [95]. This fresh discipline of investigation, known as “data science” seeks to combine statistics with fields such as data mining and interpretation. Data scientists working across different domains focus on developing statistical and machine-learning models that can generate more accurate prediction values. In the realm of machine learning, neural-network techniques are commonly employed as prediction tools.

Genomic prediction models tackle various prediction challenges by simulating real-world forecasting scenarios. To address the prediction issues encountered in GS research, several random cross-validation procedures have been developed. These procedures involve combinations of untested lines (LU), tested lines (LT), tested environments (ET), and untested environments (EU), resulting in four fundamental scenarios. For example, LU-ET (random cross-validation 1, CV1) represents the prediction of newly generated lines or cultivars that have not previously been tested [96]. A significant portion of GP research has been dedicated to developing accurate, cost-effective, nonparametric, and parametric statistical and computational models for non-phenotyped genotype prediction [25]. Breeding programs throughout the world have examined and enacted GS and GP in a variety of crops. Concurrently, substantial research efforts have resulted in the creation of novel statistical tools that incorporate environmental covariates (e.g., weather data) and genetic covariates (such as pedigree and genomic information) based on statistical–genetic prediction systems [97]. GS systems are particularly effective at capturing the non-additive genetic components that play a vital role in complex phenotypes. Complex genomic architectures and traits with low heritability are well suited to GS approaches.

In the context of forecasting features related to Fall Armyworm (FAW) and Maize Weevil (MW) resistance, several GP models have been employed, utilizing statistical and machine-learning algorithms from semi-parametric, parametric, and nonparametric approaches. As observed in previous model benchmarking reports, these GP algorithms perform differently across various features, but the differences in predictive variances are generally minor, even when large training sets are involved [91].

To accurately predict GEBVs in a variety of crops, for GS, various methods have been chosen. These include non-linear semi-parametric methods like reproducing kernel Hilbert space (RKHS) [98], linear parametric methods like ridge-regression best linear unbiased prediction (RRBLUP) [99], Frequentist methods (RRBLUP, RKHS) [100], non-linear non-parametric methods (RF) [101], Bayesian methods (BL) [102], and machine-learning methods. These methods have all demonstrated success in accurately estimating GEBVs. In GP, a TP comprising genotyped and phenotyped individuals is utilized for the computation of the combined effects of numerous markers. Based on the molecular marker profiles of non-phenotyped individuals, often referred to as the BP, the marker effects can be utilized to calculate the GEBVs [94]. Nowadays, the genomic best linear unbiased prediction (GBLUP) model is commonly utilized in plant breeding programs [103]. It enables the routine assessment of breeding values without the need for iterative procedures, owing to fixed variance components. Compared with Bayesian models, GBLUP requires less computational effort. Because of the presumption of uniformly distributed marker effects, it is frequently regarded as the approach of choice for qualities influenced by several genes.

In GS, which makes use of genomic data to predict an individual’s genetic potential for particular traits, the construction of a TP is an essential stage. Breeders try to replicate the genetic variation found in the target population when creating the TP [104]. This entails choosing a population from a variety of genetic origins, guaranteeing the sire line or family representation and taking into account the proportion of organisms in each category. It is also critical to include organisms that have accurate and trustworthy phenotypic records for the features of interest. In instances of low heritability, it is advised to make use of targeted optimization, which makes use of the test set, rather than untargeted optimization [105].

The data provide the foundation for determining the relationship between genotype and phenotype and allows for the precise prediction of breeding values. Breeders also need to prepare the genotyping method to provide enough genetic coverage while balancing economic considerations and marker density [106]. Breeders can improve genomic prediction accuracy by properly arranging the TP, allowing for more efficient selection decisions and promoting genetic advancement in the targeted traits [105].

Despite the assumptions of the GBLUP model, quantitative traits are frequently impacted by only a small subset of markers, contrary to predictions. When predicting closely related individuals, both BLUP and Bayesian models exhibit similar accuracy. However, for populations distantly related to the training and test populations, Bayesian models have demonstrated superior performance over GBLUP [107].

The accuracy of GP is influenced by genetic factors when a large number of loci affect a trait. Applying genomic prediction (GP) in breeding poses challenges on multiple levels and depends on various other factors such as:

(i) The size, genetic diversity, and relationship of the training population (TRN) to the test population (TST) are all critical factors determining genomic prediction precision. Specifically, the relationship between the cultivars in the TRN and those in the TST set, whether they are closely or distantly related, has been shown to impact the effectiveness of genomic prediction [108,109].
(ii) The heritability of the traits under selection is another crucial factor affecting the accuracy of genomic forecasting. Characters with increased heritability, which are less complex and influenced by fewer genetic factors, can be effectively predicted using a smaller number of markers with comparatively greater effects [108,109].
(iii) The accuracy or truth of genomic prediction is poorer for complicated traits that are influenced by an abundance of markers that do not exist in LD associated with QTL. Where there is a lack of correlation between markers and actual genetic factors influencing the trait, the accuracy of genomic prediction decreases [108,109].

### 7.2. Design of Statistical Models

Researchers and analysts rely heavily on the statistical models provided as an essential instrument when investigating the connection between independent and dependent variables [110,111]. The models mentioned above use statistical methods such as regression analysis and hypothesis testing to develop forecasts, identify trends, and evaluate the plausibility of correlations between data points [112]. These models help in explaining complex systems better and in predicting how those systems will behave. The process of developing statistical models includes making decisions concerning the structure of the model, selecting variables, estimating parameters, and evaluating the model’s overall performance [113,114]. The fundamental goal of statistical design is to ensure the research outputs’ precision, reliability, and authenticity while simultaneously reducing partialities and inaccuracies. This can be accomplished by reducing the number of variables included in the study.

The core concepts of validity and reliability serve as the conceptual foundations for the theoretical underpinnings of statistical design [112,113]. The idea of validity implies the degree to which a specific tool accurately measures the aspect of a construct that it was developed to analyze. The degree to which the results of multiple measures demonstrate the same level of consistency is what we mean when we talk about reliability [112,113]. It is recommended that researchers use validated and reliable instruments, establish uniform procedures for data collection, reduce the influence of bias and confounding variables, and use suitable statistical techniques to uphold the credibility and consistency of their statistical design [114,115]. Additional pillars of statistical design include the minimization of mistakes, the assurance of the ability to generalize findings, the enhancement of statistical power, and the maintenance of a balance between the internal and external validity of findings. The process of statistical design is an essential part of research. It involves the formulation of research inquiries or hypotheses, the identification of the study population or sample, the establishment of data collection methodologies [116], and the selection of appropriate statistical analysis techniques [117,118].

(a) Model Structure

Determining the model’s structure is the first stage in the statistical model creation process. When discussing a model, the term “structure” refers to the mathematical form used to represent the association among the dependent and independent variable(s) [119]. Linear models and nonlinear models are the two most popular kinds of models. The distinction between linear and nonlinear models is that the latter allows for more complex interactions between the dependent and independent variables, whereas the former only assumes a linear relationship [120,121]. Polynomial models, where polynomial curves represent the dependence and independence; exponential models, where the dependence and independence are represented by an exponential curve; and logistic models, where the probability of a categorical outcome is represented by a logistic function, are all examples of nonlinear models [122,123].

(b) Variable Selection

The next stage after the assessment of the structure of the model is to choose the independent variables that are pertinent to the study. In this process stage, you may have to identify potential predictor variables based on previous research or your theoretical understanding of the system being investigated [120,122]. Researchers may also use variable selection procedures, such as principal component analysis or factor analysis, to determine what variables are the most significant. The selection of variables is essential since having irrelevant or redundant variables in the model might result in overfitting [111,112]. In this situation, the model performs well on the data it was trained on but badly on new data.

(c) Parameter Estimation

After the model’s structure and variables have been determined, the following stage is to estimate the model’s parameters by utilizing the information gained. Calculating the coefficients that explain the association among the dependent and independent variables is a necessary step in estimating the model parameters [124,125]. Calculating the slope and intercept of the regression line is essential to linear models’ parameter estimation processes. Estimating parameters in nonlinear models can be more difficult than in linear models since nonlinear models demand the use of numerous parameters to reflect the dynamic between the variables adequately. Estimating a parameter’s value can be accomplished with several different approaches [125,126]; for example, the maximum likelihood estimation, the least squares estimation, and Bayesian inference.

(d) Model Evaluation

The final phase in the process of building statistical models is to analyze the performance of the model. Evaluation of a model involves determining how well the model matches the available data and how well it can predict outcomes based on newly acquired data. When evaluating a model, several different metrics are taken into consideration [127]. Some of these measures include R-squared (for linear models), mean absolute error or mean squared error (for regression models), and accuracy (for classification models). When determining whether or not the model is reliable, researchers may also make use of statistical methods such as bootstrapping and cross-validation [128]. The purpose of model evaluation is to check whether the model can correctly predict outcomes based on fresh data and whether it offers a satisfactory fit to the existing data [129,130].

In the GS process, the initial step involves a basic linear model, often referred to as ordinary least-squares regression (OLS). This model is represented by the equation Y = 1 nμ + Xβ + ε, where Y is the response variable, μ is the overall mean, X represents the marker genotype, β is the marker effect vector, and ε is the residual error.

However, a challenge arises when the number of markers (p) surpasses the number of observations (n), which means that there are more markers than genotype/individual/line data points. This is known as the overparameterization problem or the “big p and small n” problem (*p* >> n), especially when thousands of genome-wide markers are used [55].

To address the overparameterization issue in linear models, ridge regression (RR) is a valuable method. RR is a type of penalized regression that introduces a penalty term to the least-squares regression equation. This penalty helps to stabilize the estimates and can effectively handle situations with a large number of markers relative to the number of observations [22].

In the ridge regression (RR) model, each marker is assumed to contribute equally to the variance. However, this assumption does not hold for all traits, as different markers may have varying impacts on the trait variance. Therefore, it becomes essential to predict marker variations based on the specific genetic architecture of the trait under consideration [30].

To address this, several Bayesian models have been proposed that incorporate a prior distribution of marker effects. These models utilize the posterior distributions of marker effects and other derived quantities, such as Bayesian LASSO and Bayesian ridge regression (BRR), to conclude the model’s parameters. Another approach is the best linear unbiased prediction (BLUP), which offers different variations for marker-based models. Examples of these variations include single-step GBLUP (ssGBLUP), genomic BLUP (GBLUP), GBLUP with linear ridge kernel regression (rrGBLUP), and ridge regression BLUP (RRBLUP) [22]. Although the genomic prediction models discussed have shown good performance for traits controlled by additive genetic architecture, their effectiveness significantly declines when confronted with traits influenced by epistatic genetic architectures (Table 2).

### 7.3. Requirement for Advanced Breeding Populations for Genomics-Assisted Breeding (GAB); NAM, MAGIC, etc.

Plant genomics and molecular breeding offer a wide range of technologically advanced solutions that can handle well the issues around sustainable living and help to increase the genetic diversity of different plant species [149]. Genomics-assisted breeding and selection as a concept was rooted back in the year 2005, and since its inception has provided enormous benefits concerning plant breeding [150]. Knowledge of genomics, acquired as a result of vast sequencing and re-sequencing projects, enables us to implement genomics-aided breeding in a variety of plants. It not only provides us with improved screening methodologies based on DNA-based molecular markers or functional markers but, in turn, also helps us to implement strategic and planned plant breeding technologies [150]. Genomics-assisted breeding, a rapid method for nailing down complex traits, thereby leading to genetic improvement, eventually helps us create climate-resilient and future-secure crops, which are not only climate-smart but also economical [149,151,152].

Genomics-assisted breeding, at present, is employed to genetically improve plant species like *Coffea arabica* [153], sorghum [154], Virginia-type peanuts [155], *Triticum* sp. [156,157,158], pulses (pea, cowpea, faba-bean and lentil) [152], *Cajanus cajan* [159,160], *Cicer arietinum*, *Arachis hypogaea* [160], *Avena Sativa* [161], *O. sativa* [162], and many others.

Programs for GAB rely heavily on advanced BPs like Nested Association Mapping (NAM) and Multiparent Advanced Generation Inter-Cross (MAGIC). These populations offer priceless genetic resources and make it possible to quickly identify and apply favorable features for crop improvement [163,164]. These multiparental breeding lines have several advantages over conventional biparental breeding populations, which only handle one trait at a time, making conventional breeding tedious and time intensive [165]. Furthermore, multiparental lines have the potential to capture existing natural variations, thereby providing a high degree of polymorphisms [166] and accelerating genetic gains [167].

These advanced BPs help us dissect complex traits [168] and nail down QTLs [169], thereby supporting the enhanced generation of plant varieties by purging deleterious or unessential genes and promoting the introgression of favorable genes for the creation of superior varieties [168,170]. These breeding populations, in turn, also allow a higher degree of genetic resolution and wider polymorphisms [171].

## 8. Integrated Genomic Selection: A Unique Approach to Boost the Capacity of Genomic Selection

Cereals play an important role in our daily diet, accounting for around half of our entire dietary energy supply. With global food security concerns and difficulties created by changing climates, there is an urgency to produce better-yielding bread wheat types that are more resistant to unfavorable environmental circumstances [172].

Genomic selection is now recognized as a viable breeding technique to solve these issues, especially with regard to complex stress tolerance traits. In the instance of maize breeding, where tolerance to both abiotic and biotic stress is critical, GS has proven to be quite efficient. GS is the process of incorporating genome-wide marker data into a model to assess the genetic abilities of prospective plants for selection. For example, maize, which is the world’s fastest-growing crop and a significant contributor to the coarse grains trade, not only provides essential nutrition but also has diverse industrial applications. However, maize is very vulnerable to biotic and abiotic stressors, resulting in lower worldwide yields. As a result, increasing maize productivity has been a primary emphasis in maize breeding efforts, particularly given the problems posed by climate change [173].

To overcome the daily challenges faced due to climate change and an increasing population, an efficient and sustainable production system is required that minimizes the pressure on the ecosystem. Crop varieties with high yields and low resource requirements are essential for such production systems to address these difficulties. In the wake of uncertain worldwide food security and changing climates, breeding bread wheat with high production potential and enhanced resistance to adverse conditions, for example, is critical. However, developing such varieties is a complex task due to the genetic system governing most crop productivity traits, where the majority of genes have minimal effects. This complexity is further compounded by low heritability and high levels of epistasis. While conventional breeding methods have generated various varieties, the genetic gain per unit of time is not as substantial as with GS, although they offer the opportunity to accelerate the selection cycle [174].

Grain yield is an important factor that is impacted directly or indirectly by other qualities such as the thousand-grain weight, the number of tillers bearing panicles, the number of grains per panicle, and the number of filled grains per panicle. The effectiveness of genomic prediction for these traits has been evaluated using different TP and model types, with the accuracy of genomic prediction varying based on the trait’s heritability, TP, and models employed. While GS has been applied to only a limited number of cereals, it has demonstrated its value in enhancing tolerance to quantitatively controlled biotic stressors in cereals. Among the investigated biotic stressors, wheat has been the focus of the majority of studies on the use of GS for disease confrontation, including various rusts, *Fusarium* head blight [175], *Septoria tritici* blotch [176], tan spot [177], and *Stagonospora nodorum* blotch [178], along with disease confrontation in flax from powdery mildew [179]. Traditional breeding methods for abiotic stressors face challenges related to accuracy and repeatability. Although abiotic stress-yield QTLs have been found and transferred using molecular markers, the use of GS for abiotic stress tolerance in cereals is currently limited and requires additional investigation.

### 8.1. Speed Breeding in Genomic Selection

In plant breeding, a revolutionary method known as speed breeding has evolved. This method enables researchers to accelerate the growth of crops beyond what is possible with traditional methods [180,181]. The technique comprises raising flora in simulated environments that provide optimal circumstances, accelerating their life cycle and permitting the creation of several generations over a single calendar year.

The procedure of GS entails the investigation of plant DNA to detect particular markers that are connected to desirable qualities, such as greater productivity or resistance to diseases. Plant breeders now have access to a powerful tool that enables them to accelerate the generation of novel and improved agricultural cultivars in a substantially shorter amount of time. This tool is a combination of speed breeding and GS [182]. Breeders can quickly discover and propagate plants that exhibit desirable features by using a process called early-stage trait selection. Speed breeding and GS offer alternatives to conventional methods for early-stage trait selection, with speed breeding focused on time efficiency and GS providing a similar outcome. This makes it possible to speed up the development of crops that are well suited to certain environmental conditions as well as the demands of the market. Plant breeders gain a significant benefit from speed breeding since it enables them to rapidly evaluate innovative crop cultivars before their dissemination to agricultural producers [182]. This is a huge advantage for plant breeders. In regions where guaranteeing food security is an urgent issue, deploying speed breeding may reduce the likelihood of crop loss and increase agricultural yield [183]. The detrimental impacts of climate change on crops can also be minimized using speed breeding [184]. It may be possible for farmers to maintain or even potentially increase their agricultural productivity by creating crops that have improved their ability to adapt to changing environmental conditions [184]. However, speed breeding has its own set of drawbacks. It could lead to an increase in monocultural practices, defined by the cultivation of wide swaths of land with a single crop variety [185]. This, in turn, would increase the likelihood of disease epidemics and reduce the overall genetic variability [186]. Despite these concerns, it is incontestable that the usage of speed breeding in GS has the potential to revolutionize plant breeding and assist farmers in solving the challenges of the modern period. This is because of the technology’s ability to expedite the selection process [187].

### 8.2. Accelerating Rate of Breeding Cereals

The acceleration of breeding cycles has gained significance in creating novel and enhanced crop cultivars that can cater to the demands of the expanding populace [188]. The conventional techniques employed in plant breeding may necessitate a protracted duration of time, spanning several years or even decades, to introduce a novel variety to the market. Recent technological advancements have facilitated researchers to accelerate the process of crop development, thereby reducing the time required [189].

The objective of expediting breeding cycles is to diminish the duration between consecutive plant generations, thereby enabling breeders to expedite the process of cultivating favorable characteristics such as enhanced resistance to drought or diseases [181,190]. Through the implementation of various methodologies, scientists are now able to expedite the process of cultivating and evaluating novel plant varieties in comparison to previous practices [191].

Marker-assisted selection is a technique that has been increasingly utilized in recent times. Marker-assisted selection utilizes molecular markers, which are distinctive DNA sequences that serve as indicators for the existence of a particular gene or trait [182,192]. Through the early identification of these markers during the developmental phase, breeders can expedite and enhance the precision of trait selection. Genomic selection is a technique that is gaining momentum in hastening breeding cycles. The process of GS entails the examination of the complete genetic composition of plants [193] through the utilization of high-throughput sequencing technology [194,195]. Identifying minor genetic variations associated with particular traits enables researchers to develop more precise breeding strategies aimed at cultivating plants with desirable attributes. Agricultural biotechnology enterprises are making significant investments in these technologies due to their potential to expedite the introduction of novel varieties to the market, surpassing conventional breeding approaches [182,194]. Furthermore, accelerated breeding cycles facilitate the prompt adaptation of crops to dynamic environments and emerging hazards. This is particularly crucial in light of the obstacles presented by the phenomenon of climate change. The International Maize and Wheat Improvement Center (CIMMYT) has implemented accelerated breeding cycles to develop heat-tolerant wheat varieties that can endure elevated temperatures as a component of their climate change adaptation initiative [11,184]. Through the utilization of MAS and GS techniques, a group of researchers has successfully identified a set of genetic characteristics that can be employed to cultivate wheat varieties capable of thriving in temperatures that surpass those of conventional cultivars by up to 5 °C. The study exhibits promising prospects in mitigating the adverse impacts of heat stress on harvest, a growing concern in numerous wheat-producing areas [196,197]. Notwithstanding the vast potential of said technologies, certain apprehensions exist regarding their utilization. An area of concern is the potential ramifications of introducing genetically edited crops, which may have unforeseen impacts on the natural ecosystem [198] and the human population [199,200]. Inquiries have also arisen regarding the function of intellectual property rights in regulating the availability of cutting-edge breeding technology, which impede the ability of small-scale farmers to reap the rewards of expedited breeding cycles.

Accelerating breeding cycles is a promising innovation in plant breeding, with the potential to increase agricultural yields [201,202], bolster food security, and facilitate farmers’ ability to adapt to evolving environmental circumstances. Nonetheless, it is crucial to adopt a cautious approach toward this technology [203,204], to take into account ethical implications, and to guarantee an equitable distribution of its advantages.

### 8.3. High-Throughput Genotyping (HTG) and Genotype Imputing

In modern genetic research and analysis, high-throughput genotyping (HTG) and genotype imputing are two critical procedures. They are crucial in unraveling the intricacies of genetic variants and their effects on many traits and diseases [205]. High-throughput genotyping is a collection of technologies and techniques that enable the quick and cost-effective identification of genetic variations in large groups of people. It entails analyzing several genetic markers, such as SNPs, within the genomes of people or groups at the same time [206]. HTG platforms genotype dozens or even millions of genetic markers in a high-throughput approach using diverse methods such as microarrays and NGS [83]. The basic goal of HTG is to genotype individuals for known genetic variations in a timely and reliable manner [207]. This information can be utilized for an array of roles, including genetic association research, population genetics, evolutionary studies, and even personalized medicine [208].

Genotype imputing, on the other hand, is a computational technique that allows researchers to predict or “impute” the genotypes of individuals for markers that have not been directly genotyped but that are correlated with the genotyped markers [209]. Imputation takes advantage of LD, a non-random association of alleles at contrasting loci, to derive missing genotypes based on the patterns observed in the genotyped markers. It relies on reference panels or databases containing the genotypes of individuals who have been directly genotyped for a comprehensive set of markers [210]. One of the most significant benefits of genotype imputing is the capacity to fill in missing genotype data, increasing the density of genotyping information and improving the statistical power of genetic research. Imputation also makes meta-analyses easier by harmonizing the genotyping data from several research studies and platforms, allowing for the combined analysis of bigger datasets [211]. Furthermore, imputed genotypes enable researchers to investigate uncommon variations that were not explicitly genotyped but were imputed based on their connection with common variants [212].

HTG and genotype imputing are approaches that work well together. While HTG gives direct genotype data for a selection of markers, imputation broadens the accessible genotyping information by estimating the genotypes for additional markers. These technologies, when combined, provide a comprehensive and profitable alternative for investigating genetic variants on a large scale [205]. The application of HTG and genotype imputing has revolutionized genetic research recently. These methods have been useful in identifying genetic risk factors for complicated diseases, characterizing population genetic structures, and identifying pharmacogenetic markers for personalized therapy [213]. Furthermore, HTG and imputation have paved the way for large-scale genomic studies, including GWAS, where millions of genetic markers are analyzed across thousands of individuals [214].

### 8.4. High-throughput Phenotyping (HTP)

High-throughput phenotyping is an innovative approach that aims to accelerate the analysis and characterization of plant traits on a large scale. It involves the use of advanced technologies, automated systems, and data analytics to efficiently capture and analyze phenotypic data from plants [215]. Phenotyping is the process of measuring and evaluating observable characteristics or traits of plants, such as growth patterns, yield, disease resistance, and physiological responses [216]. Traditionally, phenotyping has been a time-consuming and labor-intensive task that is often restricted to small-scale studies due to resource constraints [217]. However, HTP has revolutionized the field by enabling the rapid and high-throughput collection of phenotypic data from a large number of plants [218].

High-throughput phenotyping platforms utilize a range of technologies and imaging techniques, including high-resolution cameras, sensors, and robotics, to capture detailed phenotypic data at various scales, from individual plants to entire fields or greenhouse setups [219]. These automated systems can perform tasks like measuring the plant height, leaf area, biomass, chlorophyll content, and even complex traits like root architecture and photosynthetic efficiency. The collected data is then processed using sophisticated algorithms, and data analytics tools to extract meaningful insights and to identify patterns or correlations [220].

One of the primary benefits of HTP in plants is its potential to speed up breeding programs. By rapidly and accurately assessing numerous plant traits, breeders can select individuals with desirable characteristics for further breeding, resulting in the production of better crop variants in a shorter time frame [191]. High-throughput phenotyping also enables the identification of novel traits or phenotypes that were previously difficult to measure manually, expanding the breeding options and enhancing crop performance [221].

In addition to breeding, HTP has broad applications in plant research and agricultural studies. It allows scientists to investigate the impacts of temperature, humidity, and nutrient availability on plant growth and development [222]. High-throughput phenotyping is utilized to investigate varied plant reactions to biotic and abiotic challenges such as diseases, pests, drought, and heat. By understanding these interactions, researchers can develop strategies to enhance plant resilience and improve agricultural productivity [221].

Furthermore, HTP plays a crucial role in phenomics research, where large-scale datasets of phenotypic information are integrated with genotypic data such as genetic markers or sequencing data. This integration enables the identification of genotype–phenotype associations and the discovery of genes or genomic regions underlying specific traits [220]. High-throughput phenotyping also facilitates the authentication of genetic markers related to desirable characters, providing valuable information for marker-assisted selection and precision breeding [223].

The adoption of HTP in plant sciences has transformed the field, opening up new opportunities for research, breeding, and crop improvement. It allows researchers to study complex plant traits, capture phenotypic variation in diverse environments, and generate large datasets for comprehensive analyses [224]. Moreover, the integration of HTP with other “omics” technologies provides a holistic understanding of plant biology and interactions with the environment [225].

High-throughput phenotyping in plants is a commanding instrument that revolutionizes the scrutiny and understanding of plant traits. By automating data collection, leveraging advanced imaging technologies, and employing data analytics, HTP enables the rapid and comprehensive assessment of plant phenotypes on a large scale. This approach has significant implications for crop breeding, plant research, and agricultural sustainability, contributing to the generation of improved crop variants and the advancement of global food security.

### 8.5. Genomic Crop Improvement by Next-Generation Sequencing (NGS)

In addition to the HTG assay, NGS has unusually accelerated the development pace of genetic techniques for staple crops. Other than technological upliftment, conceptualization is also being used to design population experiments. The concept of traditional QTL mapping is gradually changing and being replaced by second-generation sequencing of multiple alleles, traits, and recombination. A plethora of methods has been introduced, like restriction-site-associated DNA (RAD) sequencing, genotyping by sequencing (GBS), and whole genome resequencing (WGRS) for genotyping, which is a major paradigm shift in discovering and mapping DNA markers [226,227]. The data on genome-wide markers are rapidly generated and the screening of perfect phenotypes allows for a large-scale disruption of LD, which not only scans the whole genomic association and discovers novel QTL but also practices genotypic selection via GEBVs. Designing enhanced crops can be more dynamic with the use of these high-throughput molecular breeding approaches [228]. Next-generation sequencing is highly sensitive, able to detect very low-frequency variants, and interrogates millions of targets simultaneously [229]. The price of sequencing and genotyping is quickly dropping, which has an impact on the genomic breeding scenario. An emerging paradigm shift is from biparental to multiparental populations, which is easily achievable by NGS. As they provide the opportunity to explore extensive recombination and multiallelic genomes, they build an exceptional stage to practice multiparent marker-assisted recurrent selection (MARS) and GS [228]. Next-generation sequencing helps in strengthening the community-based approach of research and develops public sources like MAGIC and nested association mapping (NAM) [230]. The prior information of traditional QTL mapping is eliminated. Also, marker-assisted recurrent selection (MARS) and GS are cheaper and optimize the resources and energy to find relationships between different traits of the genome. The potency of phenotyping has limiting factors in the genetic analysis of QTLs. There is a wave of mounting demands for high-throughput screening for plant stress tolerance, such as toward abiotic and biotic stresses. The next-generation phenotyping system is trying to develop a system for wise genetic selection which will bring the hypothesis into reality for all plant species. The methods of MARS and GS, which are molecular breeding techniques, will help in extending the boundaries of genetic variation by developing superior cultivars [228].

### 8.6. Advances in Genotyping

Second-generation sequencing advancements allow the identification of innumerable SNPs in the plant genome, which are explained as follows:

#### 8.6.1. The Illumina Golden Gate Assay

This is a large-scale genotyping technique that can analyze 1536 polymorphic sites in 384 individuals. It utilizes allele-specific oligo (ASO) hybridization along with fluorescently labeled universal primers for distinguishing genotypes [231]. Several investigations have revealed the reliability of this method in scoring SNPs for genetic analysis [232]. It is also cheaper and supple enough for analyzing SNPs in large numbers [233]. The Infinium assay amplifies the whole genome, which increases the DNA amount by thousand-fold [234]. The primers specific to SNPs arrest the DNA fragments on the bead array followed by extension with hapten-labeled nucleotides. Antibodies that are labeled with fluorescent markers are then added, which detect the hapten-labeled nucleotides and give information to the user about the SNP data. This is limited to biallelic SNPs and cannot recognize indel mutations or alternative alleles. Sometimes, the deletion or accumulation of alleles deviates entities from a couple of alleles per the design of loci. Infinium classifies them as “no calls” without discriminating. It is more difficult for homologous loci to be directed in SNP probe designs rather than for highly polyploid genomes of crop plants. In this SNP probe design, there are certain limitations, as about 10–12% of the loci which passes all the specification of the design fail during the process of chip manufacturing, which means that the loci of interest is removed in the ultimate assay.

#### 8.6.2. Genotyping by Sequencing (GBS)

This is a reduced form of representation of sequencing data that uses restriction-digested genomic DNA samples and the multiplexing of samples within the same lane by “skim GBS”. This was first demonstrated in barley and maize [235]. This method has some advantages overusing a static SNP. By using raw-data mining, genotyping by sequencing can be used to accommodate a change of focus in a genome, whereas the paradigm of Infinium needs planned SNPs. It is cost-efficient and easily applied to any cereal species. Complexity reduction and enrichment of the target can also be performed to gain enough coverage in complex genomes. This method is less complicated and involves less handling of samples, the fragment size does not need to be selected, and restriction fragments with adapters are easily generated and involves reduced steps in the purification of DNA [236]. It can yield about 25,000 SNPs from one experiment, which can be utilized for the characterization of germplasm, breeding, the study of populations, and the mapping of traits [236].

#### 8.6.3. Kompetitive Allele-Specific PCR (KASP)

Kompetitive Allele-Specific PCR is a competitive allele-specific PCR-based genotyping technology. It employs allele-specific primers and a competitive allele-specific PCR technique to differentiate between genotypes. This method is inexpensive, scalable, and enables high-throughput SNP genotyping. It is widely used in plant breeding programs and genetic research [237].

#### 8.6.4. TaqMan Assay

The TaqMan test is a probe-based genotyping technology that detects SNP alleles using allele-specific fluorogenic probes. For SNP genotyping, it employs the fluorescence resonance energy transfer (FRET) concept. In SNP identification, this approach provides great specificity, sensitivity, and accuracy. It has seen widespread application in plant genetic research and breeding programs [238].

#### 8.6.5. High-Resolution Melting (HRM) Analysis

High-resolution melting analysis is a post-PCR technique for detecting SNP differences based on the melting behavior of DNA samples. It entails progressively increasing the temperature of PCR-amplified DNA fragments, observing the melting curve, and determining the SNP genotypes based on the unique melting profiles. HRM analysis is a quick, low-cost, and sensitive method for genotyping SNPs in plant genomes [239].

#### 8.6.6. MassARRAY

MassARRAY is a genotyping platform that measures the mass of allele-specific PCR products using matrix-assisted laser desorption/ionization time-of-flight mass spectrometry (MALDI-TOF MS) [240]. To identify SNP alleles, it uses allele-specific primer extension reactions and mass spectrometry analysis. MassARRAY is excellent for high-throughput SNP genotyping in plant genomes due to its high accuracy and multiplexing capabilities [233].

#### 8.6.7. Restriction-Site-Associated DNA Sequencing (RAD-seq)

RAD-seq is a method for sequencing genomic areas flanked by particular restriction-enzyme recognition sites. It entails digesting genomic DNA with restriction enzymes, ligating adapters to the fragments, and analyzing the fragments using next-generation sequencing systems. RAD-seq enables the identification and genotyping of thousands of SNPs across the genome and has been extensively utilized in population genetics, phylogenetics, and association studies [241].

#### 8.6.8. Amplicon Sequencing

Amplicon sequencing entails utilizing PCR to amplify specific target sections of the genome containing the SNPs of interest and then sequencing the amplicons. It is a targeted method for efficiently identifying and genotyping SNPs in specified genomic areas. Amplicon sequencing is frequently utilized in targeted resequencing investigations because it is both inexpensive and accurate [242].

### 8.7. Emerging Concept of Pangenomes and Super-Pangenomes

To increase the genetic variety of our crops, we must make use of the natural gene polymorphisms that exist in the populations. Globally, scientists are working to create pangenomes and super-pangenomes of different plant species, which will eventually pave the way for groundbreaking work and speed up the study of molecular breeding and plant genomics [243,244]. While reference genomes take into consideration only a single member of a given species, pangenomes are whole-genome representations at the species level that can potentially illustrate the genetic diversity of a given species [245,246]. A pangenome is created by combining genetic information from various individuals or strains of a species. It has an accessory or dispensable genome that contains genes found in only a small subset of individuals as well as a core genome that is made up of genes shared by every member of the species [247,248,249]. Therefore, efforts must be made to create pangenomes that could potentially represent more diverse polymorphic forms of a target gene to capture the full genetic diversity in terms of SNPs and structural variations and so forth [250,251]. These pangenomes are recognized as significant genetic resources that can support the development of elite crop varieties, enhance GS, and quicken breeding initiatives for the development of climate-smart crops [252,253,254].

Pangenomes of many crops have been developed to date, including *O. sativa* [244,255,256], barley [257], wheat [258], pearl millet [259], soybean [260], chickpea [247] and others like banana [261], legumes [262], etc. Efforts are being made to develop super-pangenome populations of different plant species. These include tomato [243] and *O. sativa* [263].

Recently, scientists all around the globe have started working on graph pan-genomes, which provide better visualization of the information related to the positioning of the novel sequences; they even help preserve the contiguity of the sequences as well as structural variations [244,264,265,266]. The idea of super-pangenomes, which would capture more genetic variation and depict entire genome representations at the genus level, has also gained traction. Super pangenomes are a broader term for pangenomes, which are collections of all the genes and genetic components that make up a species. A super pangenome includes both the accessory genes that differ between individuals or populations in addition to the core genes that all members of a species share. It includes the unusual or low-frequency genetic variants that are observed in a species’ genetic diversity [267].

## 9. Statistical Tools for Integrated Genomic Selection 

The main bottlenecks in GS usage are the requirement for a large number of markers and the costs associated with generating them [23]. However, current progress in high-throughput DNA sequencing (HTS) machinery has obviated the cost factors to some extent. For the integration and evaluation of massive amounts of data generated by HTS and HTP, multiple software tools are available, such as STGS, MTGS, RRBLUP, BWGS, etc. [55,268].

Ridge regression (RR) was among the first methods of GS. The RR method, in general, performs better than subset selection when prediction is of primary interest in regression problems [269]. It is comparable to BLUP when the genetic covariance between lines and their similarity in genotype space is proportional to each other [22,270]. RRBLUP was developed to utilize RR and non-additive kernels [271]. The program is based on a fast maximum-likelihood (ML) or restricted maximum likelihood (REML) approach to RR for mixed models. RRBLUP can also be used in conjunction with GWAS. In addition to residual error, it has only a single variance component. The most important function of this program is as a solver for mixed models.

### 9.1. Breed Wheat Genomic Selection

Breed Wheat Genomic selection (BWGS) is an integrated R library developed in a French cooperative private–public partnership project called Breedwheat for the easy computation of GEBVs [272]. Its two main functions are:

(1) Bwgs. cv, which performs replicated random model cross-validation on a training set of lines having genotypic and phenotypic data;
(2) Bwgs.predict, which predicts the GEBV for those lines for which the genotype is known [272].

The workflow involves:

(a) imputation of missing data;
(b) dimension reduction;
(c) GEBV estimation.

The program offers 15 choices among non-parametric and parametric methods. The most influential factors for the prediction ability are the extent of the TP as well as the minimum number of markers required for encompassing every piece of QTL information.

### 9.2. GMStool

This is also an R-based marker-set selection tool [273] for the quantitative prediction of phenotypes [274]. This selection is based on GWAS, statistics, and machine-learning methods. The tool identifies a set of SNP markers from GWAS results based on minimal *p*-values to construct an optimal marker set with an increased phenotypic prediction accuracy. The best prediction model is subsequently built with the optimal marker set. This tool has three steps.

(1) Preparation:

This needs four types of input files: a genotype information file, a phenotype information file, results from the GWAS, and a test sample list.

(2) Marker selection:

Here, the forward selection technique of regression scrutiny is applied and the sequential selection of SNP markers is performed.

(3) Final modeling.

Here, model prediction is executed using various methods such as RRB, random forest (RF), deep neural network (DNN), and convolution neural network (CNN).

### 9.3. SolGS

SolGS is open-source and runs on a Linux server. It utilizes RRBLUP and GBLUP methods for estimating GEBVs [275]. It addresses the bioinformatics and statistical challenges associated with data-intensive GS by providing an intuitive user-friendly web interface that is compatible with all major web browsers and allows for output data to be downloaded in text format. It uses R for statistical analyses and RRBLUP for statistical modeling. Model accuracy is estimated by at least a 10-fold cross-validation.

### 9.4. BGLR R-Package

The BGLR R-package is an extension of BLR, which simplifies the genomic data analyses in those regressions where the number of parameters is larger than the sample size. It relies on Bayesian regression methods that permit the integration of various parametric and non-parametric shrinkage and variable selection procedures. The software is useful for genomic as well as non-genomic applications. The algorithm is based on a Gibbs sampler and has scalar updates [276].

### 9.5. GenSel

The GenSel program was developed at Iowa State University, USA, as a part of the project entitled “Bioinformatics to Implement Genomic Selection (BIGS)”. It has been widely used to estimate molecular breeding values during animal selection trials, and it is based on the SNP for the desired phenotype. Three different input files are required for this program: a file containing genotype or marker data information, a phenotype file, and a map file. The software can be used to estimate the marker effect of a training data set using different Bayesian approaches [277]. In the case of unknown variations, Bayes C is the most effective method for estimating variances. This value can then be fitted to the Bayes B model, which is more responsive to variance components. The newest form, GenSel 4.0, utilizes MatVec, Boost, and STL libraries. Public distribution of GenSel is not permitted. Gselection contains functions for selecting important genetic markers. It then performs phenotype prediction based on fitted training data. It uses an integrated model framework developed by combining one additive and one non-additive model [278].

### 9.6. STGS

STGS, another program in R, stands for single-trait-based GS as it performs GS only for one trait. This package is a single-step solution for genomic predictions by estimating marker effects in terms of GEBVs. The program uses common methods such as RR, BLUP, ANN, LASSO, SVM, and RF.

### 9.7. MTGS

MTGS, as opposed to STGS, performs genomic predictions and GEBV computations by predicting marker effects using multi-trait data [55]. It also computes correlation effects among multiple qualities, indicating the information carried by one feature over another. It uses MTGS-based methods such as MRCE, multivariate LASSO (MLASSO), and kernelized multivariate LASSO (KMLASSO).

### 9.8. Ime4GS

Ime4GS is the successor package to the Ime4R package for fitting linear mixed models. Ime4Rs do not allow for the correlation of individuals or groups of persons, which poses a constraint in genetic studies. Ime4GS focuses on fitting LMMs with user-defined covariance structures, bandwidth selection, and genomic prediction. Hence, it can fit LMMs using different variance–covariance matrices. The program introduces random and fixed impacts, as well as accompanying variance–covariance matrices that yield fixed and random effects [279].

## 10. Issues and Challenges of Integrated Genomic Selection

The three most prevalent cereals with respect to their consumption and production are rice, maize, and wheat [280]. The sustained production of these chief cereals is imperiled by climate alterations, thus jeopardizing worldwide sustenance [281]. Genome-assisted selection techniques have been extensively used in the last two decades to facilitate crop augmentation, assisted by the establishment of high-quality genome sequence assemblies of various food crops, especially cereals [282]. Consequently, a wide assortment of genomic methods and approaches, along with immense developments in state-of-the-art techniques, are presently available for applications in crop breeding [283].

The significant genome-assisted selection approaches are GAB, NAM, and MAGIC [284], as discussed above. At the onset of employing GAB in cereals, the significant problem faced is the variation in the duration of the life cycle, which is generally long. This permits the generation of only a single generation in field environments [285]. Researchers are confronted with the cross-pollinated nature of cereal crops, which creates inconsistent heterozygosity. Consequently, it delays the crossing program, a result of which is fewer mapping populations that can be generated in comparison with other crops [286]. Moreover, the difficulties associated with incompatibility in cross-pollinated crops inhibit the establishment of inter-species population mapping [287]. Genomics-assisted breeding is further hindered by a reduced polymorphism in genes, few heritable characters, and photosensitivity [288]. As such, to avoid such limitations, MAGIC or NAM populations are being established that expedite the detection of markers that are tightly linked for several characters using high-throughput techniques [289]. Parental lines screened by genome selection may be edited before crossing [290].

Speed breeding can be achieved within just 12–18 months to hasten the editing, crossing, and selfing of generations in cereals [187]. As it relies primarily on the extension of the photoperiod, temperature regulation, and timely seed harvest for enhancing the rate of plant augmentation, the major constraint is the availability of controlled environmental conditions [32]. These settings become costly, and conjoining speed breeding with supplementary techniques necessitates surplus funds and proficiency [191]. The application of speed breeding poses a challenge in resource-deficient developing nations attributable to inadequate facilities, lack of expertise, and restricted partnerships alongside international organizations. Even if speed breeding is performed, species may show variations in genotypes, thus reducing seed yield [291]. Additionally, disproportionate photoperiods may hinder plant growth due to increased stress hormones, photo-oxidation, and elevated starch production [292].

Although innovations in genomics and phenomics are providing a better understanding of the complex biological mechanisms behind plants’ response to environmental stresses, associating genotype with phenotype is a major problem that delays the optimized use of high-throughput genomics and phenomics [293]. There is an urgent need to integrate a huge quantity of data into biologically meaningful explanations [294].

## 11. Conclusions and Prospects

As per the FAO estimate, global cereal production (2022) has shown a decline in maize, followed by rice and sorghum. The Sixth Assessment Report (AS6) of IPCC (2023) has revealed that a global temperature rise of 1.1 °C has taken place and that additional warming is expected in the coming future that will lead to an increased loss of crop biodiversity and yield. Thus, apart from conventional agricultural practices, modern rapid, efficient molecular marker-based technologies are urgently required to be scaled up for crop improvement in such a way that the emerging crop varieties show high yield as well as tolerance to multidimensional stress. Genomic selection, an advanced MAS tool in plant breeding methods, is one among several approaches to meet these requirements. It uses genome-wide markers (SNP markers) and phenotype information that make it rapid and accurate in identifying superior lines. Genomic selection achieves further higher genetic gain by integrating machine-learning-based predictive modeling in MAS that reduces the life cycle of crops. But as a limitation, the improved accuracy of GS requires an extensive period and a high developmental cost of SNP markers. Nevertheless, technological advancements such as high-throughput genotyping, phenotyping, genotype imputing, and sequencing technologies provide data at a low cost. To improve GS further, phenotypic data integration from diverse sources such as light, cameras, sensors, computers, and environmental data is being performed. The statistical tools for integrated genomic selection (BWGS, GMSTool, soIGS, RRBLUP, BGLR, GenSel, GSelection, Ime4GS, STGS, MTGS, etc.) are also being continuously improved to be more accurate and user-friendly. Currently, CRISPR (clustered regularly interspaced short palindromic repeats)/Cas9, RNA-directed nucleases (RGENs), TAL effector proteins (TALENs), and zinc finger nucleases (ZFNs) are bringing a revolution in genetics and impacting plant breeding by editing the genomes of economically important plants.

Apart from molecular technologies, ecosystem-based adaptation is being suggested by IPCC, which can help in mitigating climate change. Climate-smart crops (cereals) can be developed using GS and incorporated into sustainable agricultural practices. This will not only contribute to increased productivity and enhanced resilience but also create crop diversity and improved carbon sequestration by C4 cereal crops. There is a pressing need to convert the climate change-induced food insecurity threat into an opportunity by pushing future research priorities supported by prompt government policies that give impetus to diversifying the food basket.

## Figures and Tables

**Table 1:** A comparative chart of differences between MAS and genomic (GS) selection [30,88,89,90].

S. No.	Character	MAS	GS
**1.**	Marker number	Phenotype trait selected indirectly using genetic marker linked to the genomic region controlling trait of interest.	MAS variant based on GEBVs estimated using all the markers’ effects using a trained GS model.
**2.**	Trait nature	Effective for oligogenic traits/major QTL traits with major effects.	Effective for traits with small effects along with major effects, i.e., polygenic traits/major and minor QTLs.
**3.**	Prerequisite	Mapping and confirmation of markers connected with trait-associated QTL.	Training a good GS model in a TP utilizing genotype and phenotype data.
**4.**	Approach	It is a *targeted* approach where only markers linked to a few validated major QTLs are used to implement MAS.	It is a *holistic* approach where all the markers used in training a GS model are used to implement GS in the BP.
**5.**	Population nature	Applied on any population of a given crop if the QTL is validated, which is very rare. Relatively less effective for improving quantitative traits.	Workable on a BP that is related to or a derivative of the TP. Highly effective in improving quantitative traits.
**6.**	Implementation	To complement any of the conventional breeding strategies like MA-Backcross, MA-Pedigree, MA-Recurrent selection.	More appropriately implemented in line with development breeding.
**7.**	Genetic gain	Genetic gain per unit of time is less and much time is spent on QTL detection and validation.	Genetic gain per unit of time is relatively high as all the QTLs with major and minor effects are considered.
**8.**	Limitations	Linkage drag, background noise, and environmental instability, especially for quantitative traits.	Factors influencing prediction accuracy.
**9.**	Suitability	Complex genome, high polyploidy, heterozygosity, varied chromosome number, low/medium-density markers.	Improves the breeding efficiency and prediction, covers the entire genome, is preferred in purebred breeding across many animal species, forecasts the breeding potential of individual lines, and increases heritability estimation.

**Table 2:** Cereal Crop Model and Trait Improvements.

S.No.	Crops	Model	Trait	References
1	Maize	GBLUP	Grain yield	[131]
		RRBLUP	Grain yield	[132]
			100 kernel weight	[132]
		Bayes A, Bayes B, Bayes C, LASSO, and RKHS GBLUP and multigroup GBLUP	Grain yield	[133]
		RRBLUP and BSSV (Bayesian stochastic search variable)	Ear rot	[134]
		BLUP	Striga resistance Drought tolerance	[135]
		GBLUP	Drought tolerance	[136]
		RRBLUP and GBLUP	Water-logging tolerance	[137]
2	Barley	RRBLUP	Grain yield	[138]
		GBLUP and RKHS	Thousand kernel weight (TKW)	[139]
		GBLUP	DON resistance	[140]
3	Rice	Bayesian LASSO	Grain yield	[141]
		RRBLUP	Panicle weight	[142]
		GBLUP	Grain yield, Field grain, Field grain weight, The variance of field grain	[143]
		GBLUP, SVM, LASSO, and PLS	Field grain	[144]
		GBLUP	Field grain weight	[145]
		GBLUP, RKHS, and Bayes B	Panicle weight Nitrogen balance index	[146]
		GBLUP	Thousand-grain weight (TGW), Grain yield	[57]
		RRBLUP and LUP	Blast resistance	[147]
		GBLUP and RKHS	Drought tolerance	[148]

## Data Availability

The data are contained within the manuscript.
